# Electronic, Magnetic, Half-Metallic, and Mechanical Properties of a New Equiatomic Quaternary Heusler Compound YRhTiGe: A First-Principles Study

**DOI:** 10.3390/ma11050797

**Published:** 2018-05-15

**Authors:** Yilin Han, Yang Wu, Tingzhou Li, R. Khenata, Tie Yang, Xiaotian Wang

**Affiliations:** 1 School of Physical Science and Technology, Southwest University, Chongqing 400715, China; hanyl724@126.com (Y.H.); TZLi97@163.com (T.L.); yangtie@swu.edu.cn (T.Y.); 2 School of Materials Science and Engineering, Guilin University of Electronic Technology, Guilin 541004, China; wuyang@ihep.ac.cn; 3 Laboratoire de Physique Quantique et de Modélisation Mathématique (LPQ3M), Département de Technologie, Université de Mascara, 29000 Mascara, Algeria; khenata_rabah@yahoo.fr

**Keywords:** Heusler compounds, first-principles calculation, electronic structure, magnetism, mechanical behaviors

## Abstract

We apply First-principles theory to study the electronic structure as well as the magnetic and mechanical characteristics of YRhTiGe, a newly-designed Y-based quaternary equiatomic Heusler compound. This compound is half-metallic in nature with a ferromagnetic ground state. The total magnetic moment of YRhTiGe is 2 μ_B_ and it obeys the Slater-Pauling rule, M_t_ = Z_t_ − 18, where M_t_ and Z_t_ are the total magnetic moment and total number of valence electrons, respectively. The magnetic and half-metallic behaviors at its equilibrium and strained lattice constants have been discussed in detail. In addition, for FM-type YRhTiGe, its polycrystalline mechanical features such as Poisson’s ratio, Lame constants, Kleinman parameter and hardness, are also computed according to the well-known Voigt-Reuss-Hill approximation. We investigate the mechanical anisotropy of YRhTiGe using the directional dependences of the Young’s modulus and the shear modulus. Finally, we prove this compound is structurally and mechanically stable. This theoretical investigation provides further insight into the application of Y-based compounds as spintronic materials.

## 1. Introduction

During the last twenty years, half-metallic materials (HMMs) have been studied widely because of their possible applications as spintronic/magneto-electronic materials [1,2]. This type materials show two different spin band features [3]. The majority band structure (spin-up) shows metallic behavior, whereas the minority spin band structure (spin-down) has semiconducting (or insulating) properties with an obvious band gap around the Fermi level (E_F_). Among HMMs, one of the most attractive candidates is half-metallic Heusler magnets, because their crystal structures are comparable with zine–blende-type and rock–salt-type semiconductors and the majority of them have above room-temperature FM properties [4,5,6,7,8].

The history of Heusler compounds can be traced back to 1903 [9]. Normally, Heusler compounds can be divided into three types: The ternary Heusler materials XYZ [10,11,12,13], the full-Heusler materials X_2_YZ [14,15,16], and the equiatomic quaternary Heusler (EQH) materials XYMZ [17,18,19,20], where X, Y, M are transition-metal elements. By replacing an X with M, the Heusler system can be changed from full-Heusler to EQH. The half-metallic properties of ternary Heusler materials are usually broken by the effect of disorder [3]. And we should point that EQHs have less disorder than pseudoternary Heusler so they have lower power dissipation advantages [7].

Several theoretical investigations have been carried out on the class of rare-earth-element based EQH HMMs [21,22,23,24,25,26,27,28,29]. For example, several authors [23,27] have shown that the ScFeCrT (T = Si, Ge), LuCoCrZ (Z = Si, Ge), and YCoCrZ (Z = Si, Al, Ge, Ga) Heusler compounds are half-metallic in nature. This group of half-metallic Heusler compounds has attracted increasing attention due to its large spin-flip band gaps (even larger than 0.4 eV). Moreover, Wang et al. [28] have studied the EQH compounds, MCoVZ (M = Lu, Y; Z = Si, Ge). The most interesting obtained result by Wang et al.’s work is that the strain can be selected to control the electronic structures and physical natures of MCoVZ. By means of first-principles, Xu et al. [25] found some new nearly-spin-gapless semiconductor candidates among the 21-electron EQH systems of M [CoCr/FeMn] Z (M = Y, La, Lu; Z = Al, Ga).

In our work, a new rare-earth-element based EQH HMM, YRhTiGe, has been predicted using the first-principles. We will focus on the magnetism, electronic, and half-metallic properties of this compound. Uniform strain and tetragonal deformation will be used to examine its magneto-electronic and half-metallic properties. Moreover, we will systematically investigate the mechanical properties for ferromagnetic (FM)-type YRhTiGe, which have not been studied by other researchers. Using the Voigt–Reuss–Hill approximation, we will also calculate the elastic constants, various moduli, Poisson’s ratio, and elastic anisotropy of YRhTiGe.

## 2. Computational Method

### 2.1. Electronic and Magnetic Behavior

The calculations of the electronic structure and magnetic properties of EQH compound, YRhTiGe, were performed using the plane-wave pseudo-potential method, implemented in the CASTEP code [30] and based on density functional theory (DFT). For the exchange–correlation functional, we selected the Perdew–Burke–Ernzerhof (PBE) generalized gradient approximation (GGA) [31]. The ultrasoft pseudopotential was used to describe the interaction between ions and electrons. YRhTiGe was optimized with the following convergence parameters: 12 × 12 × 12 k-point mesh, a 450 eV cutoff energy, and a 10^−6^ eV self-consistent field tolerance.

### 2.2. Elastic Properties

For the elastic behaviors of YRhTiGe, calculations were performed in the Vienna ab-initio Simulation Package (VASP) on the basis of a projected–augmented wave (PAW) method [32]. The PBE-GGA and ultrasoft pseudopotential were selected to describe the exchange-correlation functional and the interaction between ions and electrons, respectively. For cubic structures, there are three independent elastic constants (*C*_11_, *C*_12_, and *C*_44_), which can be obtained by adding various small strain stress tensors to the grand state structures. Using the Voigt–Reuss–Hill (VRH) approximation [33,34,35] and the achieved elastic constants, we calculated the elastic moduli. The bulk modulus *B_V_* (*B_R_*) and shear modulus *G_V_* (*G_R_*) at the Voigt (Reuss) boundary for cubic materials can be calculated from the elastic moduli formula in Reference [36]. The Hill values can be seen as the arithmetic mean of the Reuss and Voigt values:

(1) $$B=\frac{\left(B_{R}+B_{V}\right)}{2}\;\mathrm{and}\;G=\frac{\left(G_{R}+G_{V}\right)}{2}.$$

The Young’s modulus *E* can be calculated by the following formula:

(2) $$E=\frac{9GB}{3B+G}$$

The Poisson’s ratio *ν* can be calculated by the following formula:

(3) $$\nu =\frac{\left(3B-2G\right)}{2(3B+G)}$$

### 2.3. Crystal Structure

**Figure 1:**
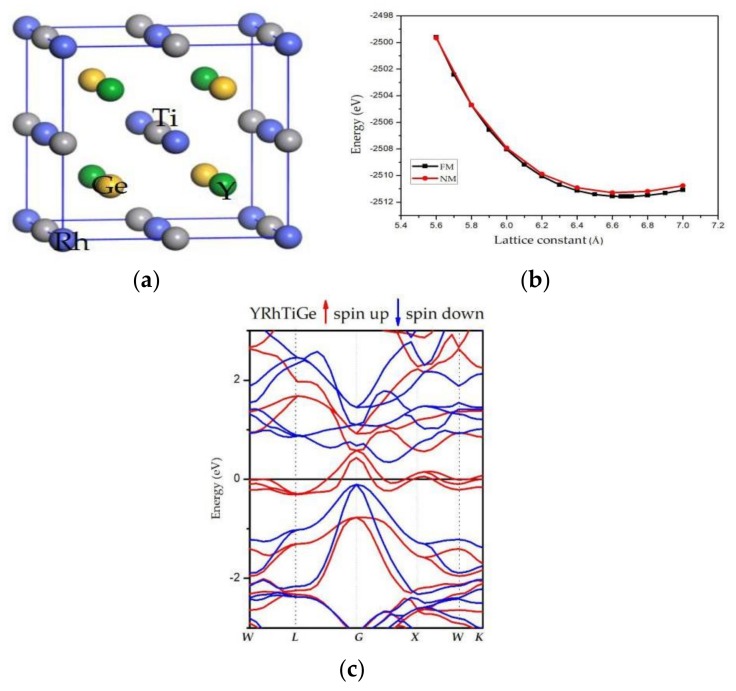
(**a**) Crystal structure (type III) of the equiatomic quaternary Heusler (EQH) compound, YRhTiGe; (**b**) Computed total energies of YRhTiGe as a function of the lattice constant in non-magnetic (NM) and ferromagnetic (FM)states, respectively; (**c**) Computed band structures of YRhTiGe at its equilibrium lattice constant.

Normally, EQH materials exhibit LiMgPdSn/Y-type structures. As shown in Table 1, it is possible for YRhTiGe compound to have three crystal structures in three different atomic positions. According to the atom-occupation rule (AOR) in EQH as well as some related works of EQH [22,25,28], transition-metal elements with fewer valence electrons tend to enter the Wyckoff site D (0.75, 0.75, 0.75), whereas transition-metal elements with more valence electrons are tended to occupy the A (0, 0, 0) and C (0.5, 0.5, 0.5) sites, respectively, and main-group elements tend to be located in the B (0.25, 0.25, 0.25) position. Thus, in this work, the Rh, Y, Ti, and Ge atoms are situated in A, B, C, D positions, respectively, as shown in Figure 1a.

### 2.4. Geometry Optimization

The total energy (TE)–lattice constant (LC) curves of YRhTiGe were obtained by minimizing the TE of this compound on various LCs for the type III structure in the FM and non-magnetic (NM) states (see Figure 1b). Due to the lower total energy, the FM state is more stable than the NM state. The computed lattice parameter for the FM-phase is 6.67 Å and the results are shown in Table 2.

## 3. Results and Discussion

### 3.1. Electronic Properties

Based on the obtained equilibrium lattice constant of the YRhTiGe compound, we discuss its electronic properties. Firstly, the calculated band structure of the FM-type YRhTiGe compound at its equilibrium lattice constant is shown in Figure 1c. One can see that the spin-up channel overlaps with E_F_; however, an indirect 0.42 eV energy gap is found near the E_F_ in the spin-down channel. Furthermore, the E_F_ sits within the spin-down band gap.

**Figure 2:**
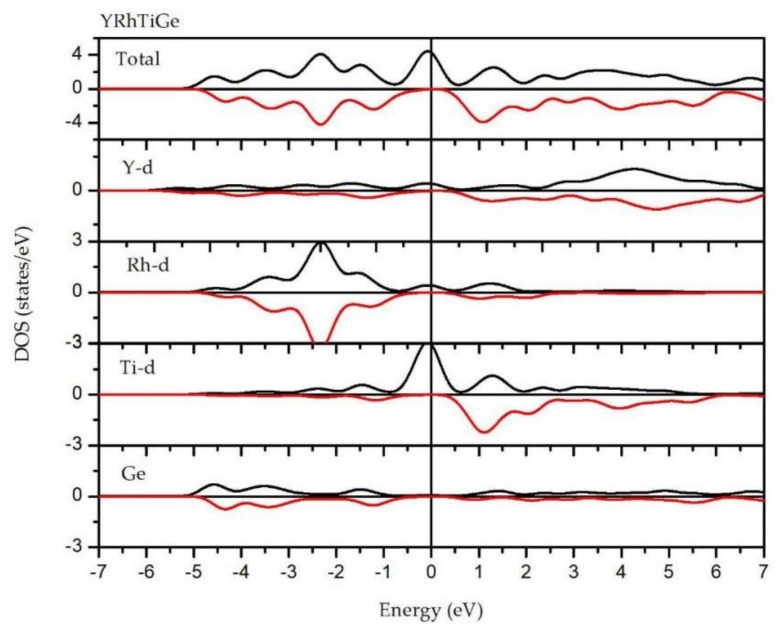
Computed total and partial density of states of YRhTiGe. The black lines and red lines represent the spin-up channel and the spin-down channel, respectively.

In Figure 2, the total and partial densities of states (DOS) of YRhTiGe compound are shown. From it, one can see that there is an open energy gap and a high DOS peak in the spin-down and spin-up directions, respectively, thus showing that YRhTiGe is a typical HMM. A very wide d-states energy distribution is determined by the strong hybridization between the d-states of the Rh and Ti atoms, varying from −1 eV to 1 eV for YRhTiGe. In the spin-down channel, the DOS near the energy gap is also dominated by the d-states of Rh and Ti atoms. The PDOSs of Y, Rh, and Ge are nearly symmetric in both spin channels. However, for the Ti atom, strong spin-splitting could be found near the E_F_, which suggests the total magnetic moment of YRhTiGe mainly comes from the Ti atom (see Table 2).

Given the total DOS of YRhTiGe, we can compute the spin polarization of electrons at the E_F_ using the equation [37]:

(4) $$P=\frac{N↑(E_{F})-N↓(E_{F})}{N↑(E_{F})+N↓(E_{F})},$$

where $N↑(E_{F})$ and $N↓(E_{F})$ are the states of the spin density at *E_F_*. The majority and minority are represented by the ↑ and ↓, respectively. The results shown in Table 3 show that YRhTiGe have a total *P* around the E_F_, reflecting the half-metallic properties of YRhTiGe.

### 3.2. Magnetism and Slater–Pauling Rule

From Table 2, the total magnetic moment (M_t_) of YRhTiGe is 2 μ_B_ at its equilibrium lattice constant. When we consider YRhTiGe to be an EQH compound, its number of valence electrons (Z_t_) is 20 and the M_t_ = Z_t_ − 18, thus the relationship between M_t_ and Z_t_ follows the Slater–Pauling curve [38]. As we discussed in Section 3.1., the spin magnetic moment of Ti is very large and contributes majorly in the total magnetic moment, while the small-spin magnetic moment of the Rh, Ge, and Y atoms make almost no contribution.

Normally, Slater–Pauling rules can be divided into three types: M_t_ = Z_t_ − 18, M_t_ = Z_t_ − 24, and M_t_ = Z_t_ − 28. These differences can be explained by the different band-gap origins for EQH compounds. More details about EQH compounds can be found in Özdoğan et al. [38]. Furthermore, different band-gap origins lead to differences in the generalized electron-filling rule. Here, we only discuss the case of M_t_ = Z_t_ − 18 with YRhTiGe as an example. Based on the calculated band structure in the spin-down direction and the band-gap origin proposed by Özdoğan et al., we can see that the band gap of YRhTiGe originates from the separated Γ_15_ and Γ_25_ states, which come from the bonded t_2g_ and antibonded t_1u_ states. Further, based on our previous work [37] on the YRhTiGe compound (Z_t_ < 21), the Fermi level is located between the t_2g_ and t_1u_ states in the spin channel, and the total number of occupied states drops to 9 (3 × t_2g_, 2 × e_g_, 3 × p, and 1 × s) with a band gap.

### 3.3. Effect of Uniform Strain and Tetragonal Deformation on the Magneto-Electronic Proprieties

The physical properties of YRhTiGe compound may undergo significant changes due to the distortion of unit cells and disorder on lattice sites, especially during film growth in experimental investigations. In this section, we study the preservation mechanism for magneto-electronic M-E and half-metallic H-M properties in the following two cases:

1. Uniform strain: For different uniform strains, although the crystal lattice maintains its cubic close-packed structure, the lattice constant tends to change.
2. Tetragonal deformation: The cubic unit cell is compressed/stretched into a tetragonal one. That is, we keep the tetragonal unit cell with volume, $V_{tetragonal}=a\times b\times c$ (a = b), and the equilibrium bulk volume, $V_{equilibrium}=a^{3}$, equal, while the c/a ratio changes from 0.80 to 1.20. Additionally, the tetragonal unit cell changes from −2%, −1%, 0%, 1%, and 2% *V_equilibrium_* have also been considered into account.

**Figure 3:**
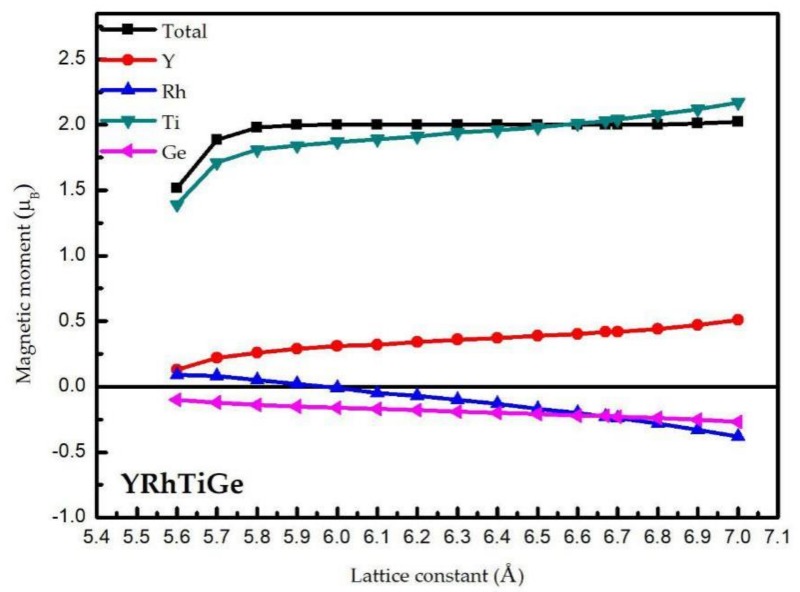
Calculated total and atomic spin magnetic moments of YRhTiGe as functions of the lattice constant.

From Figure 3, one can see that the total and atomic magnetic moments of the YRhTiGe compound can be affected by the uniform strain. The total magnetic moment of 2 μ_B_ can be maintained from 5.9 Å to 6.8 Å, which shows the H-M properties of YRhTiGe can be kept in this region. Further, in this region, with the increasing lattice constants, the values of atomic magnetic moments of the Ti and Y atoms increase while that of Rh and Ge decreases. Finally, in this region, the increased values (M_Ti_, M_Y_) and the decreased values (M_Rh_, M_Ge_) are equal. Thus, the M_t_ will be fixed at the value of 2 μ_B_.

**Figure 4:**
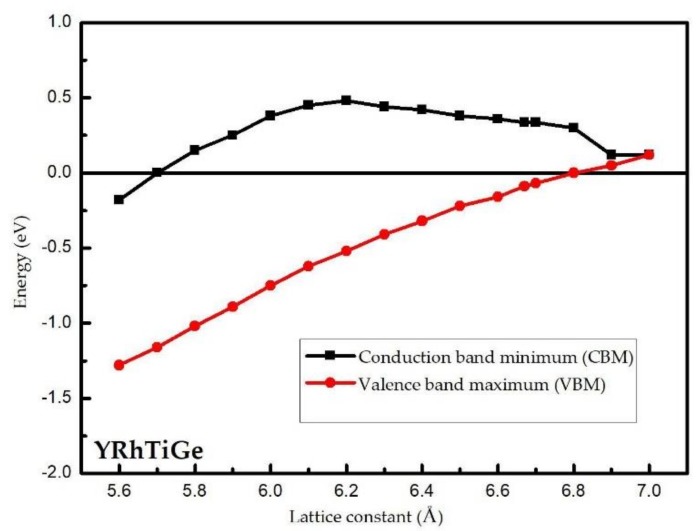
The relationship between the lattice constant and the Conduction band minimum(CBM)/Valence band maximum(VBM).

To examine the uniform strain’s effect on the electronic band structure and H-M behaviors of the YRhTiGe compound, the energy values of the conduction band minimum and the valence band maximum as a function of the lattice constant for YRhTiGe in the spin-down direction have been plotted and the results have been given in Figure 4. When the value of CBM is positive and the value of VBM is negative, YRhTiGe is an HMM. Otherwise, the EQH compound YRhTiGe will lose its H-M nature. From Figure 4, one can see that the H-M nature of YRhTiGe can be maintained in the region of 5.7–6.8 Å.

**Figure 5:**
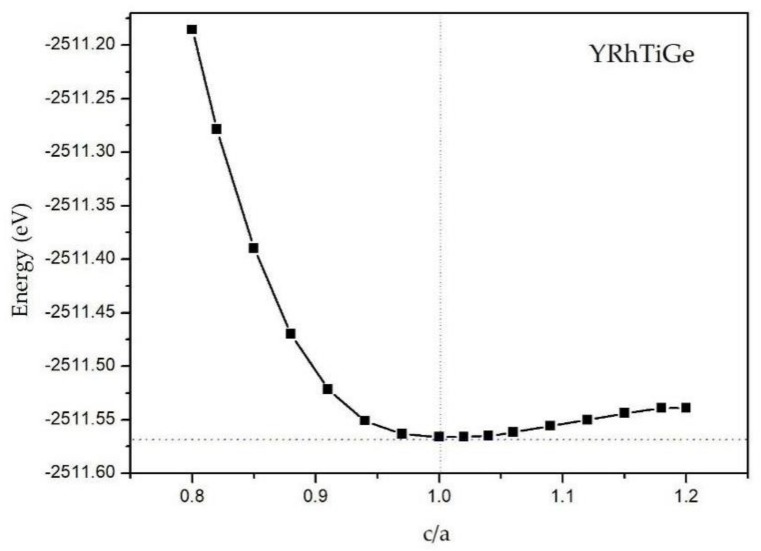
Total energies of YRhTiGe regarding the tetragonal deformation of the c/a ratio.

**Figure 6:**
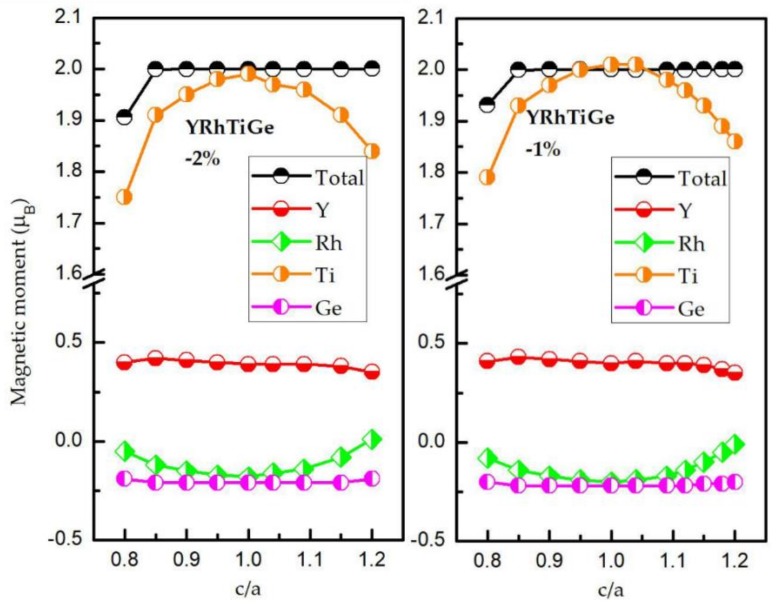

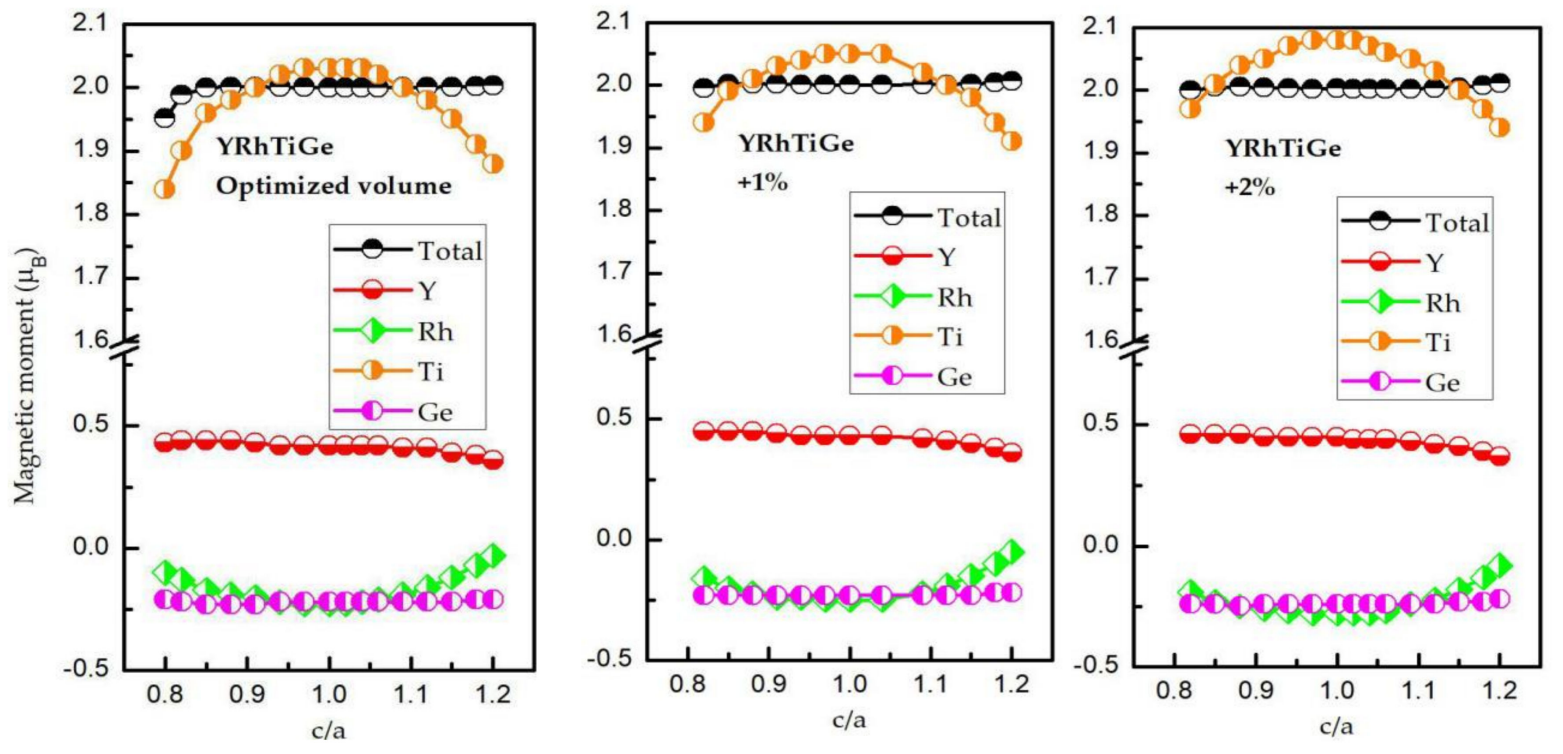
The total magnetic moments of YRhTiGe and the atomic magnetic moments of Y, Rh, Ti, and Ge atoms with contraction/expansion of the unit cell volume as functions of the tetragonal deformation of the c/a ratio.

Then, we come to study the effect of tetragonal deformation on the M-E and H-M properties of YRhTiGe. In Figure 5, the cubic structure is more stable than the tetragonal structure because the total energy of the cubic-type (c/a = 1) YRhTiGe is lower than that of the tetragonal-types. The relationship between the total/atomic magnetic moments and the c/a ratio is shown in Figure 6. The results reveal that the total magnetic moment of around 2 *μ*_B_ is preserved despite the c/a ratio changing (ranging from 0.85 to 1.15) or the increasing/decreasing of the unit cell volume. This suggests that the H-M nature of YRhTiGe exhibits low sensitivity to the tetragonal deformation effects. Furthermore, the larger degree of tetragonal deformation, the smaller (bigger) value of atomic magnetic moment of Ti (Rh). However, for the Ge and Y atoms, their atomic magnetic moments changes negligibly. On the other hand, as the tetragonal unit cell increases from −2% to 2% *V_equilibrium_*, the values of M_Rh_ (M_Ti_) decrease/increase gradually, while the atomic magnetic moments remain stable in the Y and Ge atoms.

**Figure 7:**
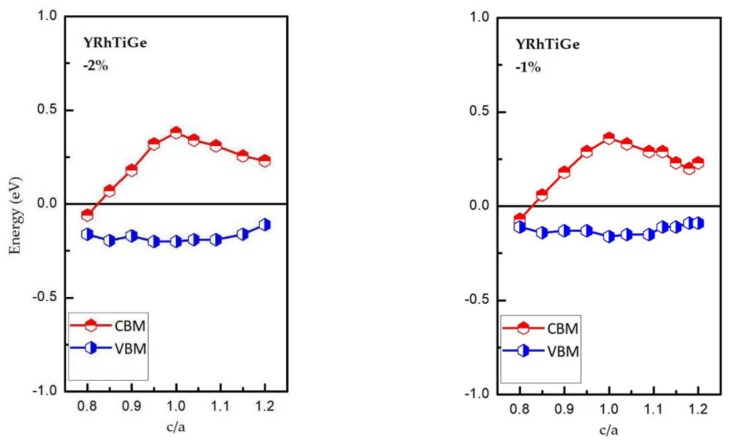

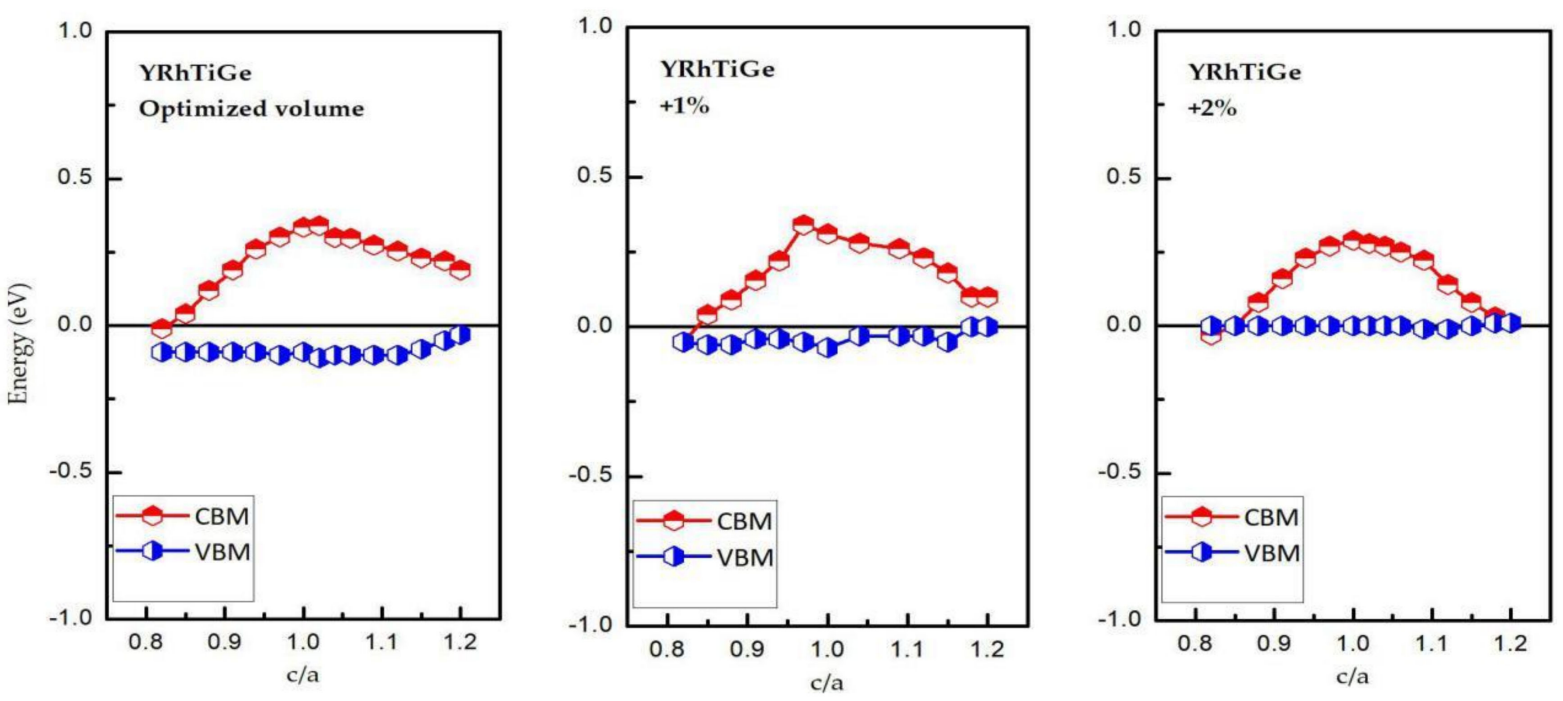
The CBM and VBM in the spin-down direction for YRhTiGe with contraction/expansion of the unit cell volume as functions of the tetragonal deformation of the c/a ratio.

**Figure 8:**
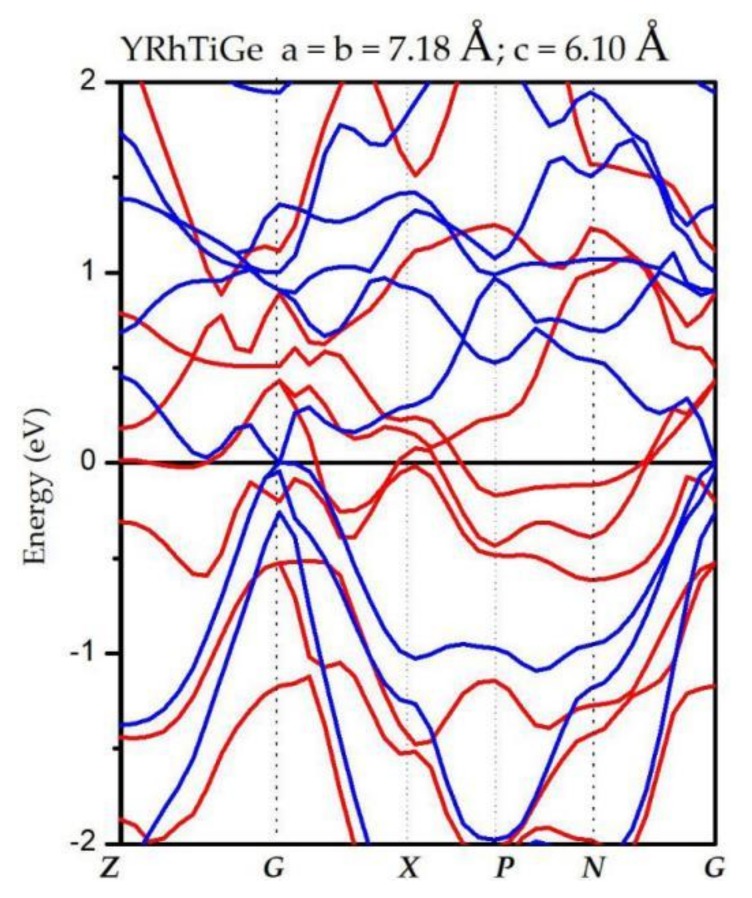
Calculated band structures of YRhTiGe at its strained lattice constant. The red lines represent spin-up channel and the blue lines represent spin-down channel.

We have also investigated the changes of the spin-down band gaps on the width and location. Figure 7 shows the values of CBM and VBM and the relationship between CBM/VBM and the c/a ratio. Obviously, for YRhTiGe, the H-M state can be maintained when c/a is in the range of 0.85 to 1.15 and the unit cell volume is changed from −2% to 2%. Furthermore, as the unit cell volume increases from −2% to 2% of the optimized volume, the CBM decreases somewhat, and the value of VBM decreases to a large extent, which leads to a reduction or even disappearance of the half-metallic band gap. Note that uniform strain or tetragonal deformation could influence the electronic structure and the physical nature of YRhTiGe. For example, as shown in Figure 8, when a = b = 7.18 Å and c = 6.10 Å, the physics feature of YRhTiGe changes from half-metallic to zero-gap half-metallic. That is, the majority spin bands are metallic, however, the semiconducting bands (minority spin) changes from non-zero gap to zero-gap (see blue lines Figure 1c and Figure 8 at G point).

### 3.4. Mechanical Proprieties

To further discovery the structural stability and mechanical properties of YRhTiGe, the elastic constants have also been computed. Our results, including elastic constants, various moduli, Poisson’s ratio, Lame constants, Kleinman parameter, and micro hardness for YRhTiGe, are given in Table 4, Table 5 and Table 6.

The *B*/*G* ratio is widely used to test the brittle/ductile property of compounds [39]. Generally, when the *B*/*G* ratio is less/more than the value of 1.75, the compound is brittle/ductile. In this work, the *B*/*G* ratio of YRhTiGe is much larger than 1.75, which shows that this EQH compound is ductile.

Usually, Lame constants (*λ*, *µ*) are used to study the hardness of polycrystalline compounds. *λ*, the first Lame constant, reflects the compressibility of compounds, and *µ*, the second Lame constant, reflects the shear stiffness of compounds, these Lame constants can be obtained using the following formulae [40]:

(5) $$\begin{array}{l} \lambda =\frac{E\nu }{\left(1+\nu )(1-2\nu \right)},\\ \mu =\frac{E}{2(1+\nu )}. \end{array}$$

Using the above formulae, the Lame constants (*λ*, *µ*) were computed as shown in Table 5.

Further, the Kleinman parameter, *ζ* is another useful parameter to show the stability of a compound for stretching and bending. The Kleinman parameter can be computed using [40]:

(6) $$\varsigma =\frac{C_{11}+8C_{12}}{7C_{11}+2C_{12}}.$$

When the value of *ζ* is close to 0, minimum bond bending is attained, and when it approaches 1, minimum bond stretching is attained. The value of *ζ* for YRhTiGe is 0.788, reflecting that the bond stretching of this EQH compound is minimal.

The formula below can be used to compute micro hardness parameter *H* [40]:

(7) $$H=\frac{(1-2\nu )E}{6(1+\nu )}.$$

We can see that the *H* value of YRhTiGe is much smaller than the *H* value of superhard materials with more than 40 GPa. Because not all the compounds are perfectly isotropic, we therefore compute the elastic compliance constants (i.e., *S*_11_, *S*_12_, and *S*_44_), anisotropy factor (*η*), direction dependences of Young’s modulus, and shear modulus for the YRhTiGe compound.

The elastic compliance constants (i.e., *S*_11_, *S*_12_, and *S*_44_) can be computed from the inverse of the matrix of elastic constants. For a cubic compound, the following formulas can be used [41]:

(8) $$\begin{array}{l} S_{11}=\frac{C_{11}+C_{12}}{\left(C_{11}-C_{12})(C_{11}+2C_{12}\right)},\\ S_{12}=\frac{-C_{12}}{\left(C_{11}-C_{12})(C_{11}+2C_{12}\right)},\\ S_{44}=\frac{1}{C_{44}}. \end{array}$$

The anisotropy factor (*η*) can be obtained either from compliance constants (i.e., *S*_11_, *S*_12_, and *S*_44_) or the elastic constants. The formulae are [41]:

(9) $$\eta =\frac{2C_{44}}{C_{11}-C_{12}}=\frac{2(S_{11}-S_{12})}{S_{44}}.$$

**Figure 9:**
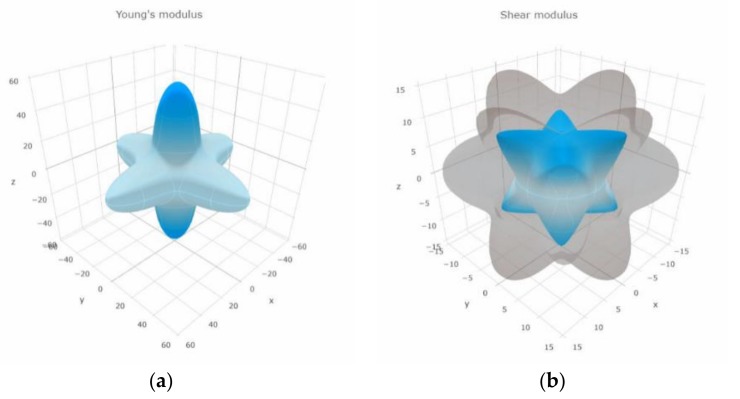
Directional dependence of Young’s Modulus (**a**) and shear modulus (**b**) for YRhTiGe.

If the anisotropy factor (*η*) is equal to 1, the compound is perfectly isotropic. In other situations, i.e., *η* ≠ 1, the compound is anisotropic. For YRhTiGe, as shown in Table 6, the anisotropy factor is 0.38, much smaller than 1, reflecting elastic anisotropy. As shown in Figure 9, we plotted the orientation dependence of Young’s modulus and shear modulus of the YRhTiGe compound, from which the anisotropy in this EQH compound can be observed. Furthermore, as shown in Table 6, one can see that the maximal values of Young’s modulus and shear modulus appears in the [001] and [111] direction, respectively.

Finally, we come to examine the mechanical stability of YRhTiGe. From Table 4, one can see that YRhTiGe is mechanically stable because the calculated elastic constants follows generalized elastic stability criteria [41]:

(10) $$C_{44}>0,\;\frac{\left(C_{11}-C_{12}\right)}{2}>0,\;B>0,\;C_{12}<B<C_{11}.$$

### 3.5. Formation and Cohesive Energies

We examined the formation and cohesive energies of YRhTiGe compound to show the structural stability of this EQH compound. The formation and cohesive energies can be obtained by the following formulae [42,43,44,45]:

(11) $$E_{formation}=E_{YRhTiGe}^{total}-(E_{Y}^{bulk}+E_{Rh}^{bulk}+E_{Ti}^{bulk}+E_{Ge}^{bulk}),$$

(12) $$E_{cohesive}=(E_{Y}^{iso}+E_{Rh}^{iso}+E_{Ti}^{iso}+E_{Ge}^{iso})-E_{YRhTiGe}^{total}.$$

where $E_{YRhTiGe}^{total}$ is the YRhTiGe’s total energy for per formula unit, and $E_{Y}^{bulk}$, $E_{Rh}^{bulk}$, $E_{Ti}^{bulk}$, and $E_{Ge}^{bulk}$ are the total energies per atom for each Y, Rh, Ti, and Ge atom in the bulk form, respectively. $E_{Y}^{iso}$, $E_{Rh}^{iso}$, $E_{Ti}^{iso}$, and $E_{Ge}^{iso}$ are the individual energies of the Y, Rh, Ti, and Ge, respectively. From the results shown in Table 3, the formation and cohesive energies of YRhTiGe are negative (−1.38 eV) and large positive (20.13 eV), respectively, indicating that this EQH compound is theoretically stable.

## 4. Conclusions

Based on PBE-GGA, we studied the electronic, magnetic, half-metallic, and mechanical properties of a newly-designed EQH compound, YRhTiGe. We found the M_t_ of this EQH HMM is 2 μ_B_, which follows the S-P rule, M_t_ = Z_t_ − 18. Two types of strain are considered in this work and we found that this compound shows low sensitivity to the uniform strain and tetragonal deformation. Furthermore, we examined the mechanical properties. The *B*/*G* ratio of YRhTiGe is much larger than 1.75, reflecting the ductility of this EQH compound. Obtained *η* indicates that the compound is anisotropic. Also, the directional dependence of the *G* and *E* moduli for the YRhTiGe compound show its anisotropic behavior. One can see that the maximal values of the *E* and *G* moduli appears in the [001] and [111] directions, respectively. Finally, we found that this compound is structural and mechanical stable.

## Figures and Tables

**Table 1:** Three possible crystal structures of YRhTiGe with different atomic positions.

Type/Atom	Y	Rh	Ti	Ge
Type 1	D (0.75, 0.75, 0.75)	B (0.25, 0.25, 0.25)	C (0.5, 0.5, 0.5)	A (0, 0, 0)
Type 2	D (0.75, 0.75, 0.75)	C (0.5, 0.5, 0.5)	B (0.25, 0.25, 0.25)	A (0, 0, 0)
* Type 3	D (0.75, 0.75, 0.75)	A (0, 0, 0)	C (0.5, 0.5, 0.5)	B (0.25, 0.25, 0.25)

Note: The type III is the most stable crystal structure on basis of the atom-occupation rule (AOR) in EQH compound YRhTiGe. * stands for the most stable phase among the three types.

**Table 2:** Total and atomic magnetic moments, and calculated equilibrium lattice constants of the type III EQH compound, YRhTiGe.

Compound	Total	Y	Rh	Ti	Ge	a (Å)
YRhTiGe	2.00	0.42	−0.23	2.03	−0.22	6.67

**Table 3:** Calculated formation energy, cohesive energy, band-gap, number of valence electrons (Zt), possible Slater–Pauling (S–P) rule, and spin polarization for the EQH compound, YRhTiGe, with type III structure.

Compound	Formation Energy	Cohesive Energy	Band-Gap	Zt	S-P Rule	P (%)
YRhTiGe	−1.38	20.13	0.42	20	M_t_ = Z_t_ − 18	100

**Table 4:** Elastic constant (*C*_11_, *C*_12_, *C*_44_), Various moduli (*B*, *G*, *E*), Poisson’s ratio (υ) for the EQH compound YRhTiGe with type III structure. We should point out that the B, G, E, and υ are all obtained on basis of the VRH approximation.

Compound	*C*_11_ (GPa)	*C*_12_ (GPa)	*C*_44_ (GPa)	*B* (GPa)	*G* (GPa)	*E* (GPa)	υ
YRhTiGe	137.2	96.5	7.74	110.1	11.53	33.4	0.44

**Table 5:** Lame constants (*λ*, *µ*), Kleinman parameter (*ζ*), and micro hardness parameter (*H*) for the EQH compound YRhTiGe with type III structure.

Compound	*λ* (GPa)	*µ* (GPa)	*ζ*	*H* (GPa)
YRhTiGe	102.38	11.536	0.788	0.389

**Table 6:** Elastic compliance constants (*S*_11_, *S*_12_, *S*_44_) and the anisotropy factor (*η*) for the EQH compound YRhTiGe with type III structure.

Compound	Compliance (10^−3^ GPa^−1^)			*η*
	*S*_11_	*S*_12_	*S*_44_	
YRhTiGe	0.017	7.18	0.129	0.3803
